# Fumigant Activity of Bacterial Volatile Organic Compounds against the Nematodes *Caenorhabditis elegans* and *Meloidogyne incognita*

**DOI:** 10.3390/molecules27154714

**Published:** 2022-07-23

**Authors:** Ali Diyapoglu, Tao-Ho Chang, Pi-Fang Linda Chang, Jyh-Herng Yen, Hsin-I Chiang, Menghsiao Meng

**Affiliations:** 1 Graduate Institute of Biotechnology, National Chung Hsing University, 145 Xingda Rd., Taichung 40227, Taiwan; diyapogluali94@gmail.com; 2 Department of Plant Pathology, National Chung Hsing University, 145 Xingda Rd., Taichung 40227, Taiwan; taudch@gmail.com (T.-H.C.); pfchang@nchu.edu.tw (P.-F.L.C.); 3 Agricultural Extension Center, National Chung Hsing University, Taichung 40227, Taiwan; jhyen@nchu.edu.tw; 4 Department of Animal Science, National Chung Hsing University, Taichung 40227, Taiwan; samchiang@nchu.edu.tw

**Keywords:** volatile organic compounds, root-knot nematodes, fumigant toxicity, *Caenorhabditis elegans*, *Meloidogyne incognita*, S-methyl thioacetate

## Abstract

Plant-parasitic nematodes infect a diversity of crops, resulting in severe economic losses in agriculture. Microbial volatile organic compounds (VOCs) are potential agents to control plant-parasitic nematodes and other pests. In this study, VOCs emitted by a dozen bacterial strains were analyzed using solid-phase microextraction followed by gas chromatography–mass spectrometry. Fumigant toxicity of selected VOCs, including dimethyl disulfide (DMDS), 2-butanone, 2-pentanone, 2-nonanone, 2-undecanone, anisole, 2,5-dimethylfuran, glyoxylic acid, and S-methyl thioacetate (MTA) was then tested against *Caenorhabditis elegans.* DMDS and MTA exhibited much stronger fumigant toxicity than the others. Probit analysis suggested that the values of LC_50_ were 8.57 and 1.43 μg/cm^3^ air for DMDS and MTA, respectively. MTA also showed stronger fumigant toxicity than DMDS against the root-knot nematode *Meloidogyne incognita*, suggesting the application potential of MTA.

## 1. Introduction

Plant-parasitic nematodes (PPNs) are economically important pests because they cause serious damage to crop production [1,2]. Studies have declared that there are over 4100 species of PPNs, causing more than USD 150 billion of destruction per year [3,4]. Among them, the root-knot nematodes, *Meloidogyne* spp., have been highlighted as the most destructive species in the world due to their wide host range, high reproduction rate, and short generation time [5,6].

For half a century, chemical nematicides have been used to control PPNs. However, abuse of the chemicals has adversely affected the environment and threatened human health [7,8,9]. Therefore, usage of some chemical nematicides is restricted in developed countries. For example, dibromochloropropane was banned from use in 1979 because of its carcinogenicity and mutagenicity, and bromomethane was phased out as a soil fumigant globally in 2015 under the directive of the Montreal Protocol on Substances that Deplete the Ozone Layer [10,11,12]. This situation has created an urge to develop novel fumigants with characteristics such as harmlessness to the environment and humans, affordability, and effectiveness to control PPNs.

Recently, nematophagous microorganisms and microbial volatile organic compounds (VOCs) have received increasing attention because of their potential in controlling PPNs [13,14,15,16]. VOCs are carbon-based low-molecular-weight (less than 300 Da) organic chemicals that are dispersed easily and have high vapor pressure at room temperature [17,18]. Microorganisms in soil or rhizosphere can release diverse VOCs such as alcohols, ketones, aldehydes, lipids, terpene, and organic acids through different biosynthetic pathways [19]. They can impose antagonist effects upon competitive species or act as signals involved in the communication among species, even from different kingdoms. For example, it has been shown that *Bacillus cereus* strain Bc-cm103 exhibited nematicidal activity against *Meloidogyne incognita* through the emission of VOCs [20], and *Pseudomonas putida* strain 1A00316 produced dimethyl disulfide (DMDS), 2-nonanone, 2-octanone, (Z)-hexen-1-ol acetate, and 2-undecanone, by which the viability of *M. incognita* was reduced [4].

This study collected VOCs from diverse environmental bacteria and evaluated their potential in controlling the model nematode *Caenorhabditis elegans* and the root-knot nematode *M. incognita*. The effects of VOCs emitted from bacteria, including *Bacillus oshimensis*, *Burkholderia cepacia*, *Burkholderia gladioli*, *Dyella japonica*, *Dyella yeojuensis*, *Pantoea ananatis*, *Pantoea eucrina*, *Pseudomonas aeruginosa* (strains 3, 6, and 10), *Pseudomonas oryzihabitans*, *Serratia marcescens*, and *Serratia rubidaea*, on *C. elegans*, were noted. The component analysis of these VOCs indicated that DMDS, emitted by a broad spectrum of bacterial strains, was the primary VOC causing the death of *C. elegans*. This study also revealed that S-methyl thioacetate (MTA) is a promising fumigant with potent fumigant toxicity to *M. incognita*.

## 2. Results

### 2.1. Fumigant Activity of Bacterial Strains toward C. elegans

**Figure 1:**
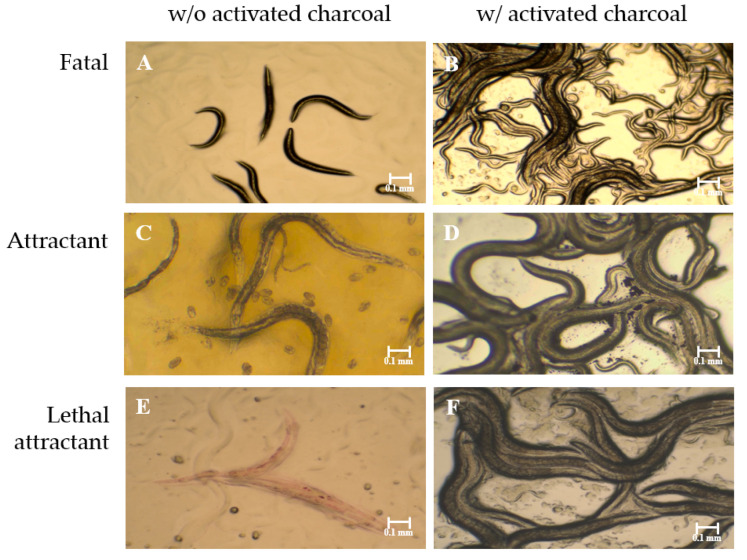
**Exemplified fumigant activity of VOCs emitted by screened bacteria toward *C. elegans*.** (**A**) The worms were immobilized after fumigation with VOCs emitted by *B. gladioli* grown on LB agar. (**B**) Same treatment as (**A**) but with activated charcoal. (**C**) The worms were lured to *D. yeojuensis* cultivated on LB agar and laid eggs. (**D**) Same treatment as (**C**) but with activated charcoal. (**E**) The worms were lured to *S. marcescens* cultivated on LB agar and died afterward. (**F**) Same treatment as (**E**) but with activated charcoal.

*C. elegans* and each of the screened bacteria were cultivated in separate compartments in a two- or three-compartment Petri dish. Later on, the effect on the nematode exerted by the bacterial VOCs was examined under a light microscope. The effective VOCs could exhibit a fatal effect, as the worms were stiff and immobilized; an attractant effect, as the worms crawled from the compartment with Escherichia coli OP50, the food source of *C. elegans*, to that with the screened bacterium; and a debilitating effect, as the worms’ motion became slow and restricted. None of the effects showed up if activated charcoal was included in the compartmental dishes, confirming the causative role of VOCs (Figure 1).

The fatal effect was derived from *B. gladioli*, *B. cepacia*, *B. oshimensis*, *P. ananatis*, *P. aeruginosa* (strains 3 and 6), *S. rubidaea*, and *S. marcescens*, when these bacterial strains were cultivated on LB agar. Previously, we showed that the composition of VOCs emitted by *B. gladioli* might be different, depending on the culture medium [21]. Therefore, the fumigant activity of VOCs emitted by *B. gladioli* and *B. cepacia* cultivated on potato dextrose (PD) agar was also examined. VOCs produced under this culture condition exerted a debilitating effect on *C. elegans*, rather than a fatal effect. To determine if glucose itself was sufficient to attenuate the toxicity of VOCs, the nematode was then challenged by *B. gladioli* cultivated on 2% glucose- or fructose-supplemented LB agar. Indeed, the fatal effect on the nematode disappeared due to the addition of glucose, but not fructose, in the LB agar. This result indicates that glucose could suppress the production of certain toxic VOCs.

The attractant effect was derived from *D. yeojuensis* and *S. marcescens*. The lure of these two bacterial strains was so strong that the worms actually crawled over the wall dividing the compartments. The worms started to lay eggs after feasting on *D. yeojuensis*. Nonetheless, the worms devouring *S. marcescens* became stiff and immobilized.

The debilitating effect was derived from *D. japonica*, *P. eucrina*, *P. aeruginosa* strain 10, and *P. oryzihabitans*, which were cultivated on LB agar, and *B. gladioli* and *B. cepacia*, which were cultivated on PD agar. The agility of the worms in this category was significantly reduced.

All the above observations indicate that the fumigant activity varied with not only the bacterial strains, but also the culture mediums. Knowing the composition of VOCs emitted from every bacterial culture might help to establish the probable links between specific VOCs and the fumigant activity.

### 2.2. Identification of the VOCs

VOCs emitted from every bacterial culture were extracted by headspace solid-phase microextraction (SPME) and the composition was analyzed by gas chromatography–mass spectrometry (GC-MS). Among the peaks observed on the GC chromatogram, only those that met the two criteria were considered as significant VOCs emitted by the tested bacterium in this study: first, the area under a peak must be more than 2% of the total peak areas; and second, the peak must not be detected in the medium control group. In other words, the peaks that also appeared in the control groups were excluded (Figure S1).

The GC chromatograms of *B. gladioli* cultivated on LB and PD agar were different (Figure S2). DMDS was the main compound, accounting for 56.92% of the total peak areas when *B. gladioli* was cultivated on LB agar. However, DMDS was not emitted by *B. gladioli* cultivated on PD agar. The fumigant toxicity of DMDS toward nematodes has been repeatedly presented in the literature [3,22], therefore, the inability to produce DMDS in such a large quantity should explain why the fatal effect was lost when the culture medium was changed from LB to PD agar. Surprisingly, carbon disulfide (11.4% of the total peak areas) was produced by *B. gladioli* cultivated on PD agar. There has been a long history of using carbon disulfide as a disinfectant against nematodes. Nonetheless, only a debilitating effect was observed in this case. The production of carbon disulfide at a relatively low level might account for this observation. To understand whether glucose suppresses the production of DMDS, *B. gladioli* was cultivated on 2% glucose- or fructose-supplemented LB agar. The GC chromatograms demonstrated that DMDS was still the main compound when the bacterium was cultivated on fructose-supplemented LB agar (Figure S3). By contrast, no DMDS was emitted when the bacterium was cultivated on glucose-supplemented LB agar (Figure S3). Clearly, the presence of glucose in the culture medium suppressed the production of DMDS in *B. gladioli*. Once again, the production of DMDS correlated with the fatal effect exerted by *B. gladioli* cultivated on fructose-supplemented LB agar.

The collection of VOCs emitted by *B. cepacia* was also analyzed by GC-MS (Figure S4). DMDS was the major component (65%) when this bacterium was cultivated on LB agar. The vast production amount of DMDS should account for the fatal effect. As with *B. gladioli*, DMDS disappeared from the collection when the bacterium was cultivated on PD agar (Figure S4). Consequently, VOCs emitted *by B. cepacia* cultivated on PD agar exerted only a debilitating effect on *C. elegans*.

VOCs emitted by *D. yeojuensis* held a remarkable attraction for *C. elegans*. The worms literally moved from the compartment with *E. coli* OP50 to that with *D. yeojuensis* and laid eggs afterward (Figure 1C). VOCs produced by *D. japonica* weakened the agility of the worms. A comparison of the VOCs produced by these two *Dyella* species revealed several common VOCs, such as 2-butanone, 1-butanol, 3-methyl-1-butanol, and 2-methyl-1-butanol, and a couple of species-specific VOCs (Figure S5).

VOCs emitted by *B. oshimensis* cultivated on LB agar (pH 10.5) included DMDS (44.9%), 2-butanone (8.1%), 3-methylfuran (4.7%), MTA (2.7%), and dimethyl silanediol (2.3%) (Figure S6). The production of DMDS should be responsible for the fatal effect imposed by this alkaliphilic Gram-positive bacterium.

VOCs emitted by *P. ananatis* included indole (28%), 2-pentanone (18%), (p-hydroxyphenyl)-phosphonic acid (14%), 3-methyl-1-butanol (8.4%), and other minor components (Figure S7). VOCs emitted by *P. eucrina* included 1-methoxy-2-propanol (21%), 3-methyl-1-butanol (13%), 5-chloro-1-pentene (7.6%), 2-methyl-1-butanol (6.3%), carbon disulfide (3.6%), and DMDS (2.3%) (Figure S7). The minor amounts of carbon disulfide and DMDS could be the reasons for the reduced agility of *C. elegans*. VOCs emitted by *P. oryzihabitans* consisted of DMDS (22%), 3-methyl-1-butanol (15%), ethylene cyclopropane (12%), methanethiol (11%), 2-butanone (7.9%), and other minor components (Figure S7).

Three *P. aeruginosa* strains, isolated from different sources, were tested in this study. The composition of VOCs produced by strains 3 and 6 were similar (Figure S8), both containing DMDS (34% and 25%, respectively), whereas strain 10 did not emit DMDS (Figure S8). Production of DMDS should be the main cause of the fatal effect exerted by strains 3 and 6. It was noted that dimethyl sulfide was produced by strains 6 and 10 (39% and 25%, respectively). Dimethyl sulfide was reported to extend the longevity of *C. elegans* by alleviating the accumulated oxidative damage in aged worms [23]. Additionally, a recent report declared that dimethyl sulfide did not affect *M. incognita* [24].

The *S. marcescens* strain screened in this study was red-colored due to the renowned red pigment prodigiosin, while the *S. rubidaea* was white. The compositions of VOCs emitted by these two Serratia strains were relatively simple compared with the others. *S. rubidaea* emitted DMDS (52%), dimethyl trisulfide (32%), and 1-[1-methyl-3-(1-methylethyl)-1H-pyrazol-5-yl]-ethanone (6.9%) (Figure S9). VOCs from *S. marcescens* contained DMDS (65%), anisole (8.9%), and 2-butanone (3.4%) (Figure S9). Obviously, the production level of DMDS should account for the fatal effect on *C. elegans*. Intriguingly, the worms would also crawl from the compartment with *E. coli* OP50 to that with *S. marcescens* before they were killed by the vapor of DMDS. The presence of red dots inside the gut of the worms suggests that the bacterium was eaten before the death of the worms (Figure 1E).

### 2.3. Fumigant Activity of Pure VOCs

**Figure 2:**
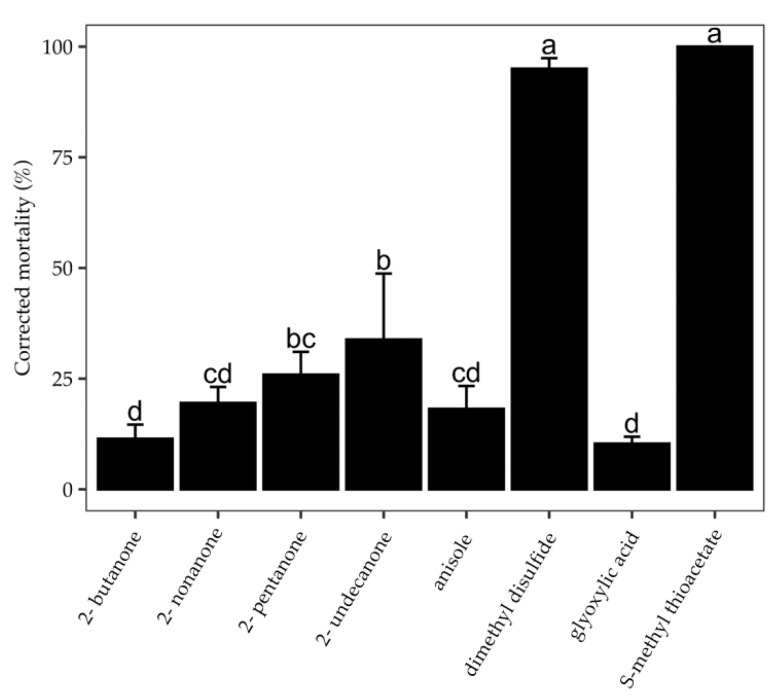
**Fumigant activity of VOCs at a dosage of 40 μg/cm^3^ air against *C. elegans* for 24 h**. The values show the corrected mortality rate of the tested VOCs compared with the control group. Bars indicate the standard error (SE) of the means. Data were analyzed by one-way ANOVA. Different letters indicate significant differences of means ± SE. The worms used in the treatments were mainly L1s with only a few adults.

The screen described above indicates a close link between DMDS and the fatal effect of bacterial VOCs on *C. elegans*. In addition to DMDS, several compounds such as glyoxylic acid, 2-pentanone, 2-nonanone, 2-undecanone, and MTA were present preferentially in the VOC collections with a fatal effect. All these compounds are commercially available; therefore, their authentic effects on *C. elegans* were tested in this study. Anisole, 2,5-dimethylfuran, and 2-butanone were also included in the test for comparison. The amount of the chemicals inside a two-compartment Petri dish was 4 mg dissolved in a total of 200 μL of 0.3% Tween-20 aqueous solution (equivalent to 40 μg chemical per cm^3^ air). The corrected mortality of *C. elegans* under the fumigation condition for 24 h was calculated (Figure 2). Among the tested compounds, MTA had the strongest fumigant toxicity, which caused 100% mortality. DMDS caused a 95.01% mortality rate at the same dosage. However, the mortality caused by other VOCs, except 2,5-dimethylfuran, was in the range of 10–35%. Fumigation with 2,5-dimethylfuran did not increase the mortality of the nematode compared with the control group (not shown in Figure 2), although the vapor of 2,5-dimethylfuran did suppress the growth of several plant pathogenic fungi [21].

Furthermore, the mortality caused by a serial dilution of DMDS and MTA was determined. The dosages in a range of 5–40 μg/cm^3^ air were used for DMDS, and 0.625–40 μg/cm^3^ air for MTA. The probit analysis based on the relation between mortality and dosage suggested that the values of LC_50_ of DMDS and MTA were 8.57 and 1.43 μg/cm^3^ air, respectively (Table 1).

**Figure 3:**
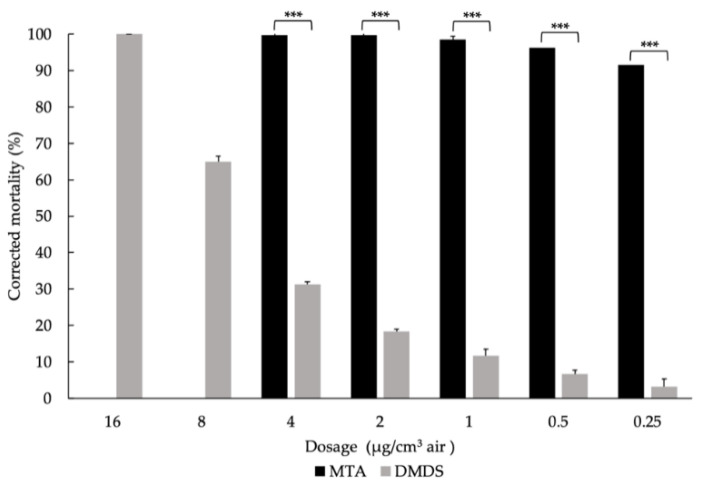
**Comparison of the mortality caused by DMDS and MTA at different dosages against the root-knot nematode *M. incognita* for 24 h.** Bars indicate the standard error (SE) of the means. Statistical comparisons were performed with Student’s *t*-test. *** denotes *p*-value < 0.001. The worms used in the treatments were J2s.

The fumigant toxicity of DMDS and MTA was tested against M. incognita using a 1 L plastic bottle. The range of dosage was 0.25–16 μg/cm^3^ air for DMDS and 0.25–4 μg/cm^3^ for MTA (Figure 3). The results demonstrated that fumigation with MTA resulted in over 90% mortality at all tested dosages. However, the mortality caused by DMDS was 3.16, 6.64, 11.63, 18.41, 31.21, 64.98, and 100% at the dosages of 0.25, 0.5, 1, 2, 4, 8, and 16 μg/cm^3^ air, respectively. The results indicate that MTA exhibited stronger fumigant toxicity to the root-knot nematode than DMDS.

## 3. Discussion

This study aimed to explore novel bacterial VOCs that might be useful in agricultural practice to control RKNs. Initially, the effects on *C. elegans* of the VOCs emitted from more than 40 bacterial strains were examined. Although *C. elegans* is not a parasite, it has been widely used as a model nematode for biocontrol experiments because it is easily maintained in the laboratory and the information obtained from *C. elegans* might apply to parasitic nematodes [25]. Thirteen strains of the screened bacteria emitted VOCs that exerted a fatal effect, a debilitating effect, or an attractant effect on *C. elegans*. We attribute the fatal effect mostly to a sufficient production level of DMDS, except in the case of *P. ananatis*. This study also confirmed that aliphatic ketones such as 2-undecanone and 2-pentanone are detrimental to *C. elegans*, although their effectiveness is not as strong as DMDS. Indole was the most abundant VOC emitted by *P. ananatis*, followed by 2-pentanone. It was reported that indole in the medium could modify the behaviors of *C. elegans* by acting as a chemoattractant at low concentrations or a virulent factor at high concentrations [26]. Therefore, the combined actions of indole and 2-pentanone could account for the fatal effect of *P. ananatis*-emitted VOCs.

VOCs emitted by *P.* *oryzihabitans* only exerted debilitating effects on the agility of *C. elegans*, although the faction of DMDS was up to 22% in the VOC collection. The possible reasons for this observation are speculated as follows. First, a fraction of 22% referred to the relative abundance, but accurate concentration, of DMDS in this VOC collection. Perhaps the concentration was not high enough to cause a fatal effect on the nematode. Second, other components in this collection might offset the activity of DMDS. These speculations remain to be verified in the future.

The behaviors of *C. elegans* responding to the VOCs emitted by *D. yeojuensis* and *S. marcescens* were intriguing. The worms seemed to be lured by the two bacterial strains based on the observation that they crawled, over the plastic barrier, from the compartment grown with *E. coli* OP50 to the neighboring compartment with *D. yeojuensis* or *S. marcescens*. The feast on *D. yeojuensis* further promoted the worms to lay eggs. Comparisons among the VOC collections emitted by the various bacterial cultures could not give conceivable explanations for the behaviors. Each component in the VOC collection emitted by *S. marcescens* could be found in other VOC collections, which exhibited no attractant activity. Therefore, the lure for *C. elegans*, in this case, is still vague. The VOC collections emitted by *D. yeojuensis* and *D. japonica* share many common components, such as 2-butanone, 1-butanol, 3-methyl-1-butanol, and 2-methyl-1-butanol. 10-methyl-1-undecene was a VOC specifically emitted by *D. yeojuensis* in this study. Therefore, it is tempting to speculate that 10-methyl-1-undecene acted as either an attractant or trigger for egg-laying behavior. Nonetheless, this hypothesis needs to be verified in the future.

We confirmed that glucose suppresses the production of DMDS in *B. gladioli*. L-methionine-γ-lyase is the key enzyme for the biosynthesis of volatile sulfur compounds in *Brevibacterium linens* [27]. This enzyme catalyzes the cleavage of L-methionine into α-ketobutyric acid, methanethiol, and ammonium; subsequently, methanethiol could be further oxidized to generate dimethyl sulfide, DMDS, and dimethyl trisulfide [28]. Therefore, it is reasonable to hypothesize that the suppression of DMDS by glucose is mediated through catabolite repression of L-methionine-γ-lyase.

In this study, MTA was identified in VOCs emitted by *B. gladioli*, *B. cepacia, B. oshimensis*, and *P. oryzihabitans.* MTA was also detected in many plant species such as *Allium cepa* and has been used as a flavoring additive. We uncovered that MTA exhibited strong fumigant toxicity to *C. elegans* as well as *M. incognita*. Parallel experiments indicate that MTA has higher fumigant activity at lower doses than the commercial biopesticide DMDS. In the literature, MTA was reported to inhibit the growth of plant pathogenic fungi except oomycetes [29]. While our work was in progress, the nematicidal activity of MTA, emitted by *Bacillus aryabhattai*, against *M. incognita* was reported for the first time [30]. Given its natural status and potent fumigant toxicity, the potential of MTA as a soil fumigant to control RKNs deserves further evaluation.

## 4. Materials and Methods

### 4.1. Chemicals and Mediums

DMDS (99%), glyoxylic acid (50% *w*/*w*), and 2-butanone (99%) were purchased from Alfa Aesar (Ward Hill, MA, USA). Anisole (>99%), 2-pentanone (>99%), MTA (>99%), 2-undecanone (>98%), and 2-nonanone (>98%) were purchased from Tokyo Chemical Industry (Tokyo, Japan). 2,5-dimethylfuran (99%) was purchased from Acros Organics (Geel, Antwerp, Belgium). Tween-20 was purchased from Sigma-Aldrich (St. Louis, MO, USA). Yeast extract and tryptone were purchased from Cyrusbioscience (Taipei, Taiwan) and Zymeset (Taipei, Taiwan), respectively. Potato extract was purchased from Formedium (Hunstanton, UK).

### 4.2. Bacterial Strains

A dozen species of bacteria, isolated from diverse environments, were screened for their nematicidal activity toward *C. elegans*. They were *B. gladioli*, *B. cepacia*, *D. yeojuensis*, *D. japonica*, *B. oshimensis*, *P. ananatis*, *P. eucrina*, *P. oryzihabitans*, *S. rubidaea*, *S. marcescens*, and a few *P. aeruginosa* strains (strain numbers 3, 6, and 10). *E. coli* OP50, the food source of *C. elegans*, was provided by Dr. Yi-Chun Wu (National Taiwan University, Taiwan).

In general, LB broth (5 g/L yeast extract, 10 g/L tryptone, and 10 g/L NaCl) was used for the cultivation of the bacteria, unless otherwise indicated. PD broth (4 g/L potato extract and 20 g/L glucose) was also used in the culture of *B. gladioli* and *B. cepacia*. Agar (15 g/L) was included in the medium when an agar plate was prepared. The culture temperature was 28 °C.

### 4.3. C. elegans and M. incognita

*C. elegans*, obtained from Dr. Yi-Chun Wu (National Taiwan University, Taiwan), was reared at room temperature on nematode growth medium (NGM) agar (2.5 g/L peptone, 3 g/L NaCl, 1 mM MgSO_4_, 1 mM CaCl_2_, 25 mM KH_2_PO_4_, 5 mg/L cholesterol, and 17 g/L agar). *E. coli* OP50 was cultivated on NGM agar according to previously described protocols [31].

An amount of 25-day-aged water spinach (*Ipomoea aquatica*, No.1 Taoyuan, Known-You seed Co., Ltd.) was inoculated with *M. incognita* (200 juveniles per plant). After 30 days of inoculation, the infected roots of water spinach were rinsed to remove the attached soil, and the revealed root knots were collected. Root knots were smashed in a mortar with sterilized water to expose egg masses. The smashed samples were then dosed with bleach (10% of final concentration) and homogenized for 1 min. The homogenized samples were filtered through a homemade collector (top filter, 250 mesh; bottom filter, 500 mesh). The egg masses that remained in the bottom filter were then rinsed 2 to 3 times with sterilized water to remove the bleach. The egg masses were then cultivated in the hatching tank (with a 500 mesh filter) in the dark under 25 °C. J2 nematodes were collected after 3 days of cultivation.

### 4.4. Fumigant Activity of Bacterial Strains against C. elegans

Bacterial strains with nematicidal activity via fumigation were screened in a two-compartment Petri dish according to the method as described in [32] with some modifications. A 50 μL aliquot of bacterial culture (OD600 ≈ 1) was transferred onto the agar medium in one compartment, while *E.coli* OP50 was transferred onto the NGM agar in the other compartment. The plate was incubated at 28 ℃ for 24 or 48 h. A chunk of agar containing about 200 *C. elegans* was placed on the NGM agar afterward. After being sealed tightly with parafilm (Bemis, Neenah, WI, USA), the plate was incubated at 25 °C for another 24 h. The viability of the nematodes was examined under a light microscope (Leica DM500, Leica Microsystems, Germany). A three-compartment Petri dish was used to confirm the fumigant activity of bacterial VOCs by placing activated charcoals in the third compartment. The activated charcoal could adsorb volatile compounds, consequently blocking their fumigant activity.

### 4.5. Extraction of Bacterial VOCs by SPME

A 125 mL Erlenmeyer flask containing 5 mL of agar medium was seeded with 50 μL of bacterial culture. The flask was sealed tightly with parafilm and incubated at 28 °C for 3 days. An SPME needle filled with a 75 μm CAR/PDMS SPME fiber (Supelco, Bellefonte, PA, USA) was preconditioned with helium at 280 °C for 30 min before use. Then, the SPME needle was used to pierce through the parafilm layers and held for 30 min to adsorb the VOCs accumulated in the headspace of the flask. VOCs emitted by the blank medium in the flask were also extracted and served as the background control.

### 4.6. Identification of VOCs by GC-MS

GC-MS was conducted to identify the VOCs. After the extraction of the VOCs, the SPME fiber was inserted into the injection port of the GC-MS instrument (QP2010 SE, Shimadzu Corp., Kyoto, Japan) equipped with an RTx-5MS column (30 m length × 0.25 mm diameter × 0.50 μm thickness). The carrier gas was helium with a flow rate of 1 mL/min. GC oven temperatures were programmed as follows: 40 °C for 5 min, 40 to 120 °C at a rising rate of 3 °C/min, 120 to 180 °C at 4 °C/min, 180 to 280 °C at 20 °C/min, and held at 280 °C for 5 min. The identity of VOC was suggested by comparing the mass spectrum of the substance with the GC/MS system data bank of the National Institute of Standards and Technology (NIST20). Experiments were conducted at least twice for each of the bacterial cultures.

### 4.7. Fumigant Activity of VOCs against C. elegans and M. incognita

Fumigant toxicity of purchased VOCs against *C. elegans* was conducted in a two-compartment Petri dish, in which a small glass bowl (2 cm ID) was placed in each of the compartments. Nematodes (n ≈ 200), suspended in ddH_2_O, were placed in one glass bowl, meanwhile, a 200-μL aliquot of diluted VOC in 0.3% Tween-20 aqueous solution was placed in the second glass bowl. An amount of 0.3% Tween-20 aqueous solution was used in the control group. The plates were tightly sealed with parafilm and incubated at 25 °C for 24 h.

The fumigant toxicity of DMDS and MTA against *M. incognita* was tested in a 1 L plastic container. A Petri dish, which contained approximately 300 nematodes, was placed in the bottom of the container, and 200 μL of DMDS or MTA at the indicated concentrations was added to a cotton ball stuck to the interior wall of the container. The container was capped, sealed, and incubated at 25 °C for 24 h.

The number of mobile and immobile nematodes was recorded with the aid of a stereomicroscope. The experiment for each of the VOCs was conducted twice, each with three replicates. The mortality rate was calculated based on the following formula: mortality rate (%) = (number of dead nematodes/total nematodes) × 100. The percentages of dead nematodes were corrected by eliminating natural death in the control group according to the Schneider–Orelli formula [33] as follows:

Corrected mortality % = [(mortality percentage in treatment − mortality percentage in control)/(100 − mortality percentage in control)] × 100%.

### 4.8. Statistical Analysis

The relationship between the mortality of *C. elegans* versus dosage of VOC was analyzed by probit analysis, from which the values of LC_50_ and LC_95_ (dosages causing 50% and 95% mortality, respectively) could be predicted. Statistical analyses of the mortality caused by various VOCs were performed using RStudio version 4.2.0. Data were analyzed by one-way ANOVA, and mean values of different treatments were compared with Tukey’s test, with different lowercase letters indicating significant differences between treatments (*p* < 0.05). Statistical comparisons specifically between DMDS and MTA were performed with Student’s *t*-test, and significant differences were determined according to a threshold of * *p*  <  0.05, ** *p*  <  0.01, and *** *p*  <  0.001.

## 5. Conclusions

The remarkable fumigant toxicity of MTA against *C. elegans* and *M. incognita* suggests a potential role of this compound as a biocontrol agent against agricultural pests. However, to confirm this potential, the activity of MTA must be also tested under greenhouse and field experiments against a wide range of pests. Moreover, the results obtained from *Burkholderia* strains indicates that growing bacteria on different mediums could change the nematicidal effects and the types of VOCs. More investigations are needed to understand the detailed mechanism.

## Figures and Tables

**Table 1:** **LC_50_ and LC_95_ of DMDS and MTA against *C. elegans* for 24 h fumigation**.

Compound	n	LC_50_ (95% CI) (µg/cm^3^ Air)	LC_95_ (95% CI) (µg/cm^3^ Air)	Slope ± SD	Chi-Square
DMDS	6000	8.57 (7.26–9.86)	42.25 (32.95–60.18)	2.37 ± 0.23	1.46
MTA	9600	1.43 (1.10–1.81)	4.58 (3.27–8.39)	3.26 ± 0.44	5.66

n: Approximate number of total *C. elegans* used in the test for each of the compounds.

## Data Availability

Not applicable.

## Supplementary Materials

The following supporting information can be downloaded at https://www.mdpi.com/article/10.3390/molecules27154714/s1: Figure S1: The GC chromatograms of VOCs emitted by LB agar and PD agar. Figure S2: The GC chromatogram of VOCs emitted by *Burkholderia gladioli* cultivated on LB agar (A) or PD agar (B). Figure S3: The GC chromatogram of VOCs emitted by *Burkholderia gladioli* cultivated on 2% fructose-supplemented LB agar (A) or 2% glucose-supplemented LB agar (B). Figure S4: The GC chromatogram of VOCs emitted by *Burkholderia cepacia* cultivated on LB agar (A) or PD agar (B). Figure S5: The GC chromatogram of VOCs emitted by *Dyella yeojuensis* (A) or *Dyella japonica* (B). Figure S6: The GC chromatogram of VOCs emitted by *Bacillus oshimensis* cultivated on LB agar. Figure S7: The GC chromatogram of VOCs emitted by *Pantoea ananatis* (A)*, Pantoea eucrina* (B), or *Pseudomonas oryzihabitans* (C). Figure S8: The GC chromatogram of VOCs emitted by *Pseudomonas aeruginosa* strain 3 (A)*,* 6 (B), or 10 (C). Figure S9: The GC chromatogram of VOCs emitted by *Serratia rubidaea* (A) or *Serratia marcescens* (B). Figure S10: Display of bacteria in the fumigant activity test using two- or three- compartment Petri dishes. A chunk of agar with *C. elegans* was placed later onto the center of the compartment on which *E. coli* OP50 was grown.
