# Supplementary material for: Fumigant Activity of Bacterial Volatile Organic Compounds against the Nematodes Caenorhabditis elegans and Meloidogyne incognita
Source: Molecules. 2022 Jul 23;27(15):4714. doi: 10.3390/molecules27154714 (PMC9330711; doi:10.3390/molecules27154714)
Supplement: Supplementary file 1 [file molecules-27-04714-s001.zip › molecules-1808795-supplementary.pdf]

A. LB agar

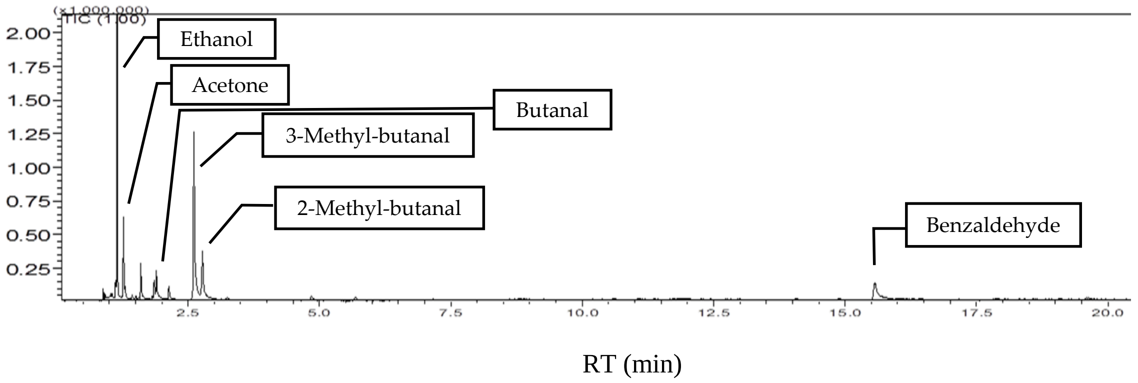

B. PD agar

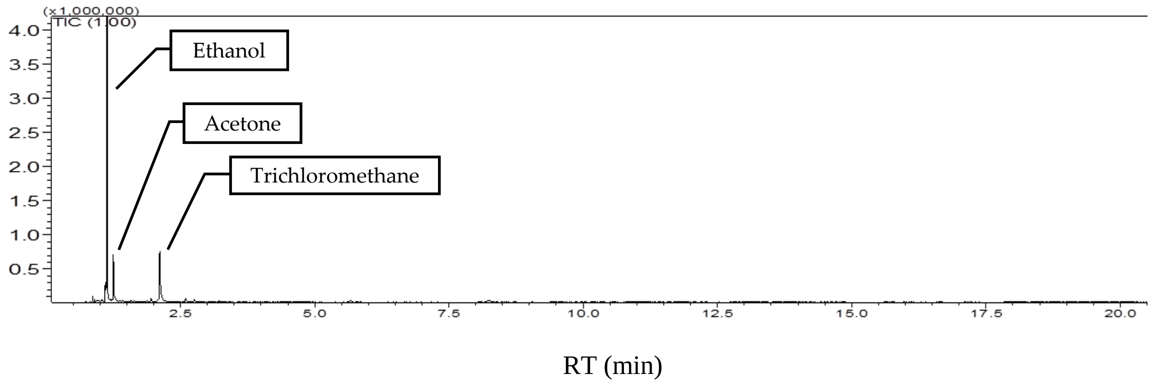

Figure S1. The GC chromatogram of VOCs emitted by LB agar (A) or PD agar (B).

(A) *B. gladioli* on LB agar

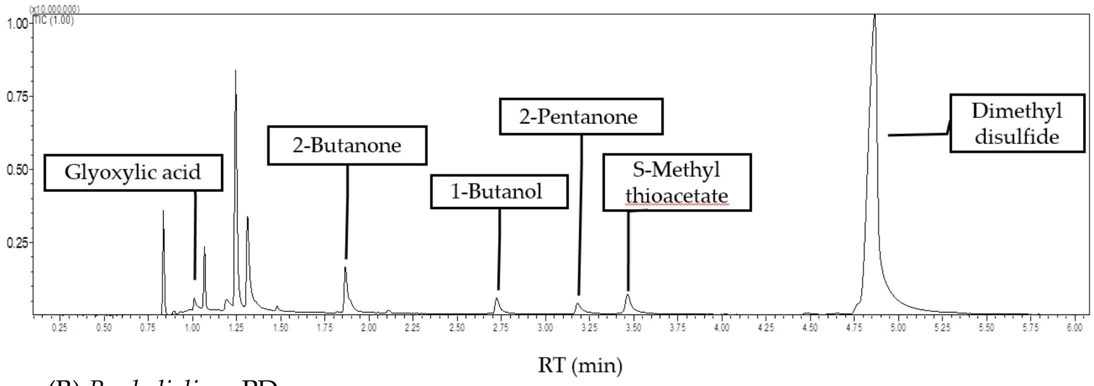

(B) *B. gladioli* on PD agar

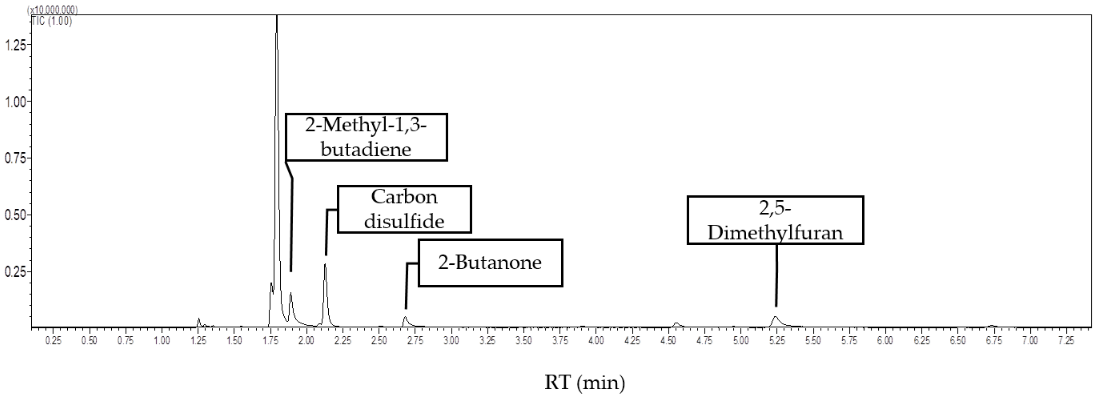

Figure S2. The GC chromatogram of VOCs emitted by *Burkholderia gladioli* cultivated on LB agar (A) or PD agar (B). The peaks without labels represent components that were also detected in the medium control.

(A) *B. gladioli* on 2% fructose-supplemented LB agar

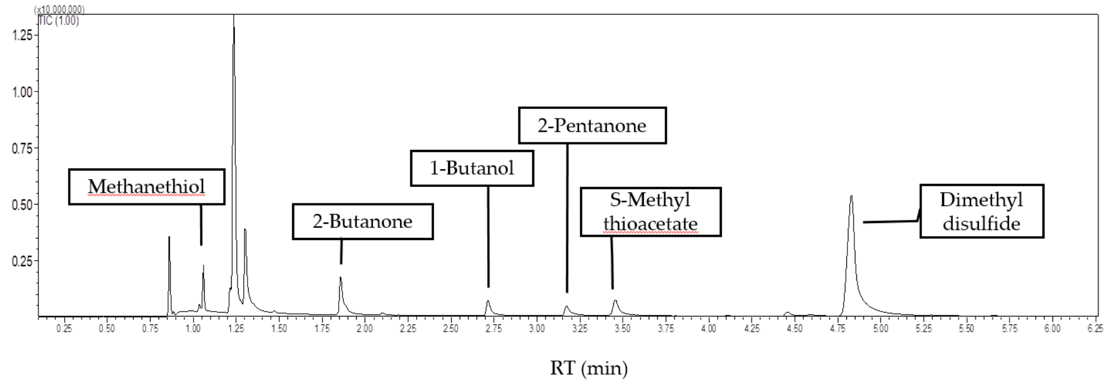

(B) *B. gladioli* on 2% glucose-supplemented LB agar

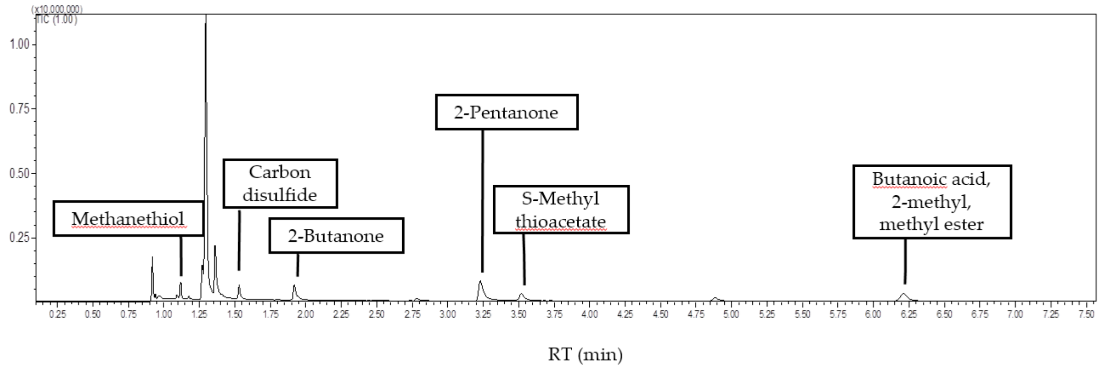

Figure S3. The GC chromatogram of VOCs emitted by *Burkholderia gladioli* cultivated on 2% fructose-supplemented LB agar (A) or 2% glucose-supplemented LB agar (B). The peaks without labels represent components that were also detected in the medium control.

(A) *B. cepecia* on LB agar

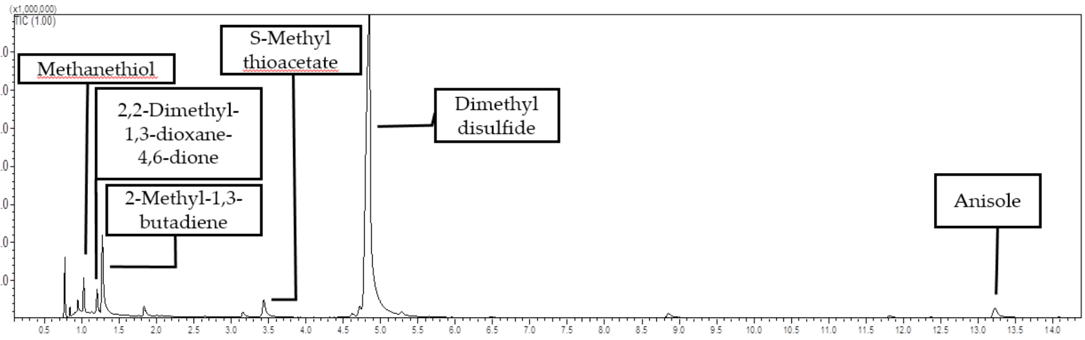

RT (min)

(B) *B. cepecia* on PD agar

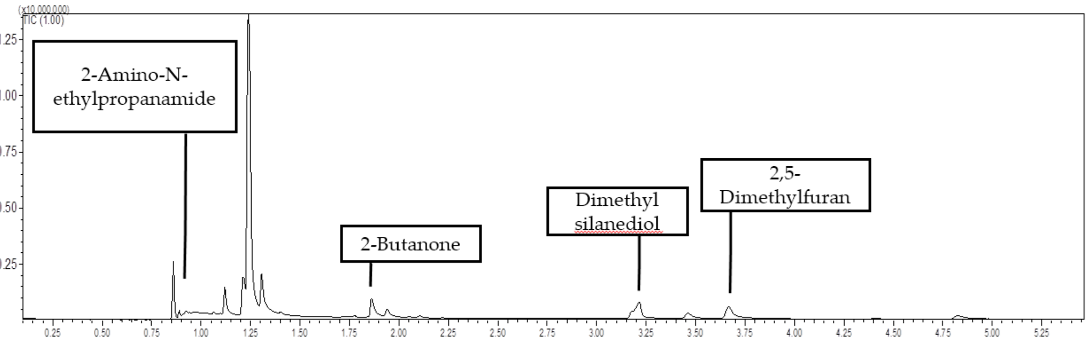

RT (min)

Figure S4. The GC chromatogram of VOCs emitted by *Burkholderia cepacia* cultivated on LB agar (A) or PD agar (B). The peaks without labels represent components that were also detected in the medium control.

(A) *D. yeojuensis* on LB agar

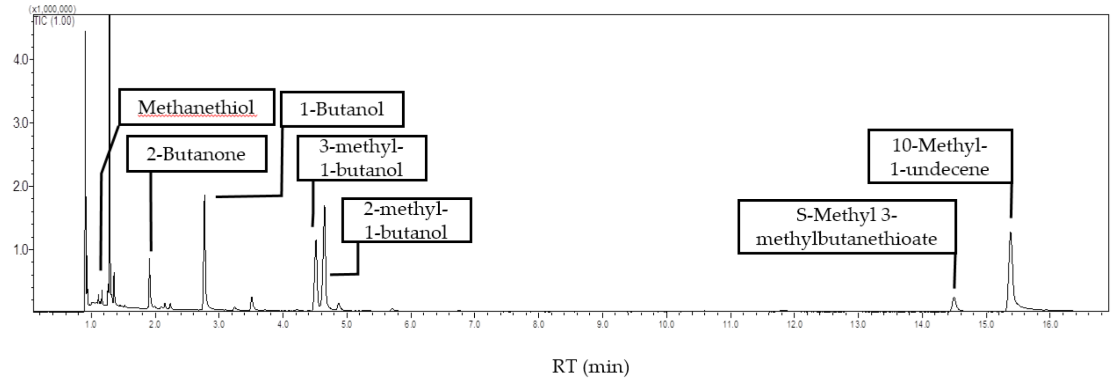

(B) *D. japonica* on LB agar

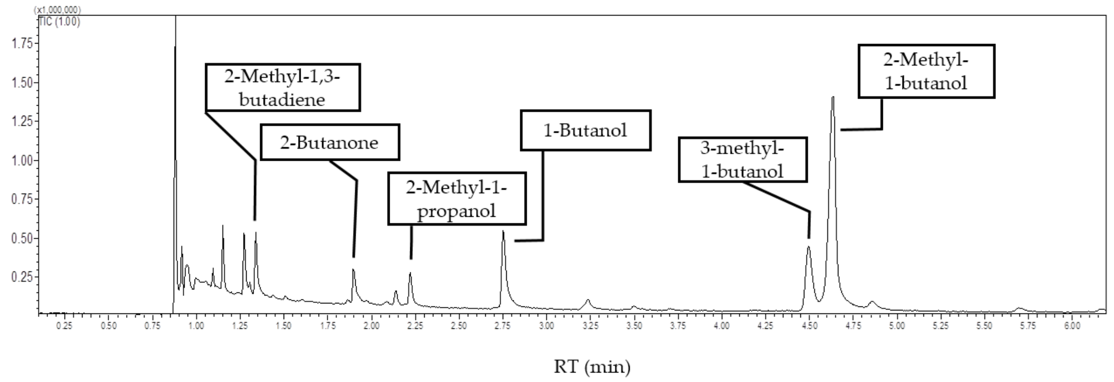

Figure S5. The GC chromatogram of VOCs emitted by *Dyella yeojuensis* (A) or *Dyella japonica* (B). Both strains were cultivated on LB agar. The peaks without labels represent components that were also detected in the medium control.

B. oshimensis on LB agar

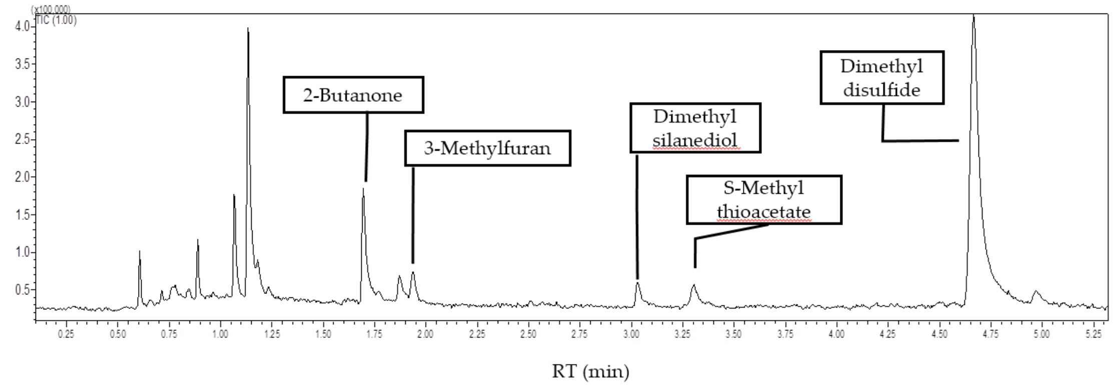

Figure S6. The GC chromatogram of VOCs emitted by *Bacillus oshimensis* cultivated on LB agar. The peaks without labels represent components that were also detected in the medium control.

(A) *P. ananatis* on LB agar

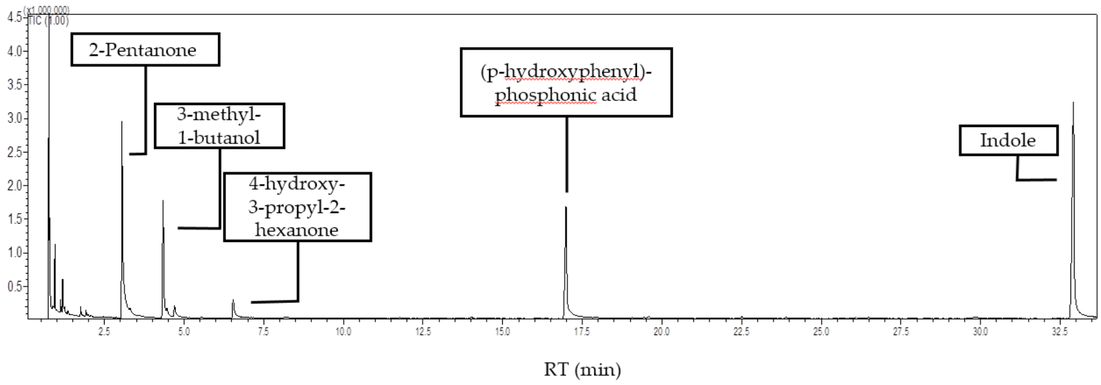

(B) *P. eucrina* on LB agar

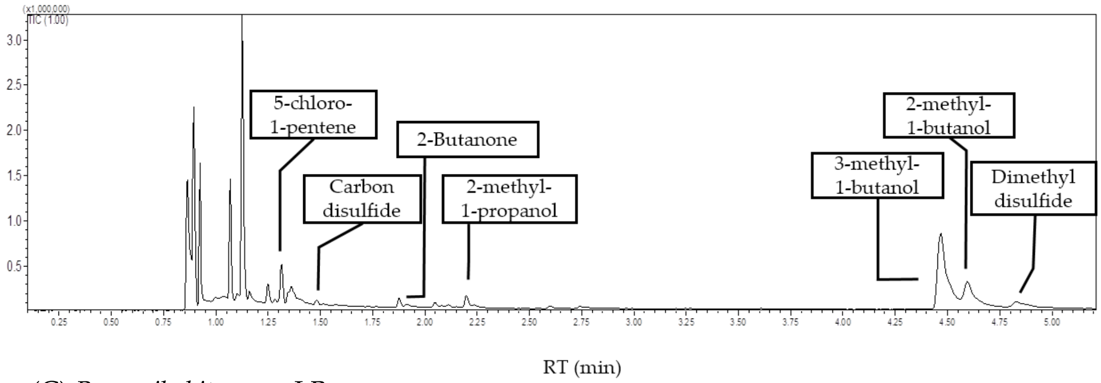

(C) *P. oryzihabitans* on LB agar

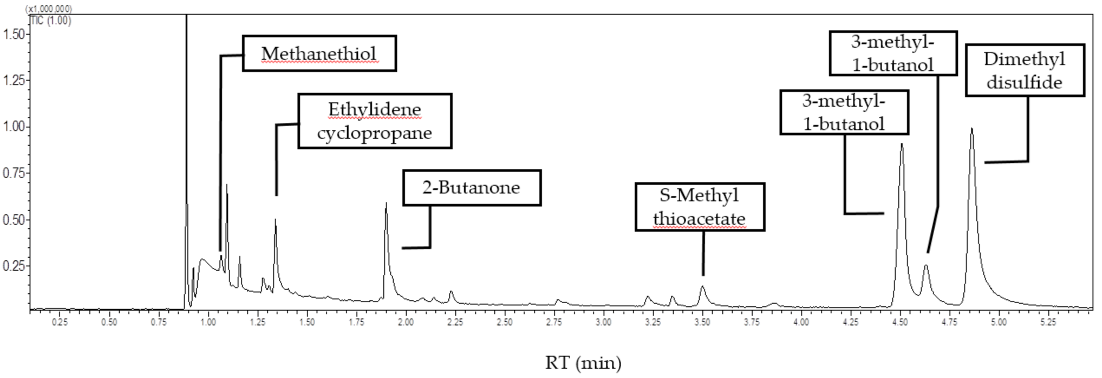

Figure S7. The GC chromatogram of VOCs emitted by *Pantoea ananatis* (A), *Pantoea eucrina* (B), or *Pseudomonas oryzihabitans* (C). All the strains were cultivated on LB agar. The peaks without labels represent components that were also detected in the medium control.

(A) *P. aeruginosa* #3 on LB agar

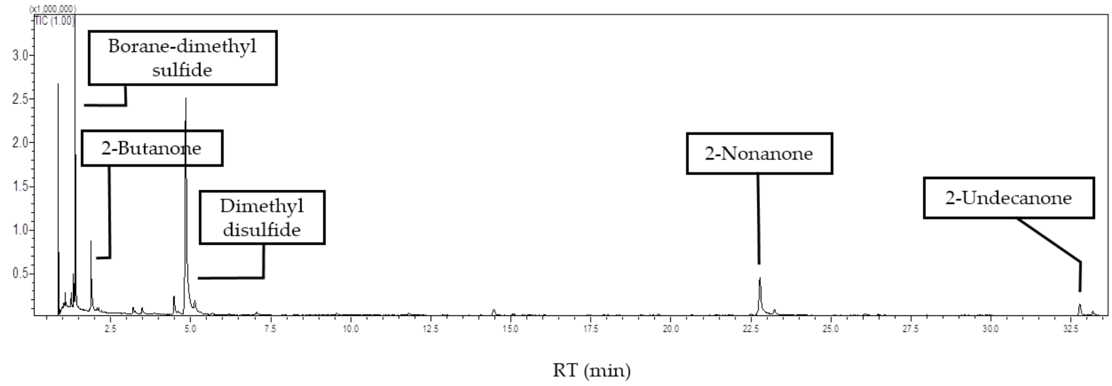

(B) *P. aeruginosa* #6 on LB agar

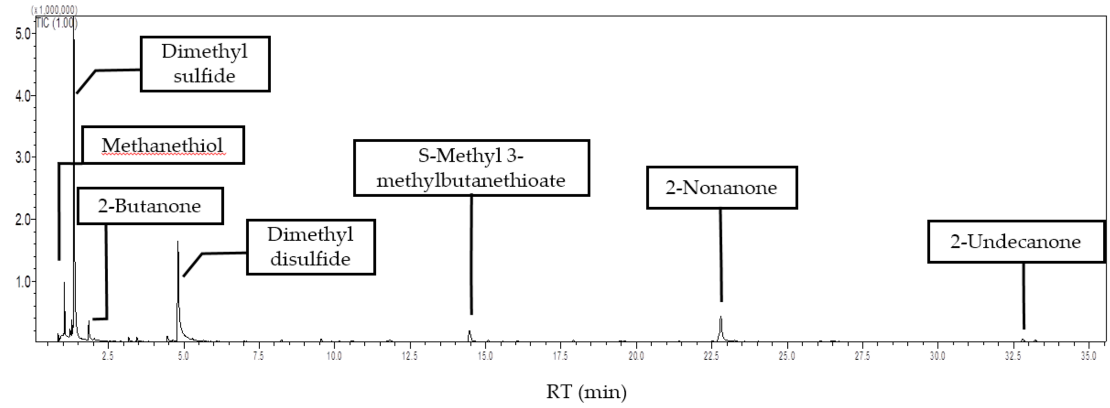

(C) *P. aeruginosa* #10 on LB agar

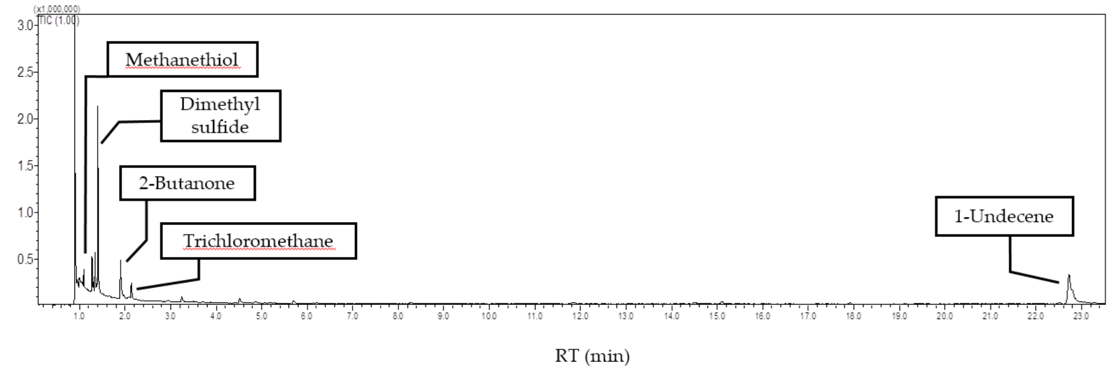

Figure S8. The GC chromatogram of VOCs emitted by *Pseudomonas aeruginosa* strain #3 (A), #6 (B), or # 10 (C). All the strains were cultivated on LB agar. The peaks without labels represent components that were also detected in the medium control.

A. S. rubidaea on LB agar

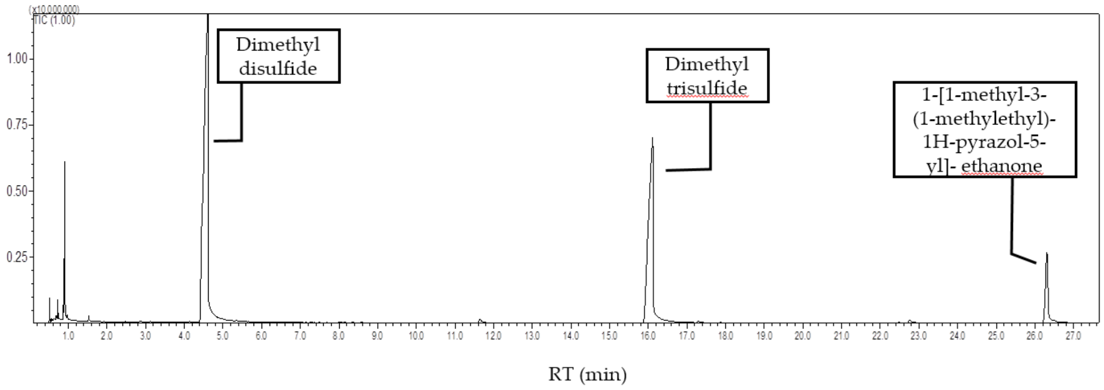

B. S. marcescens on LB agar

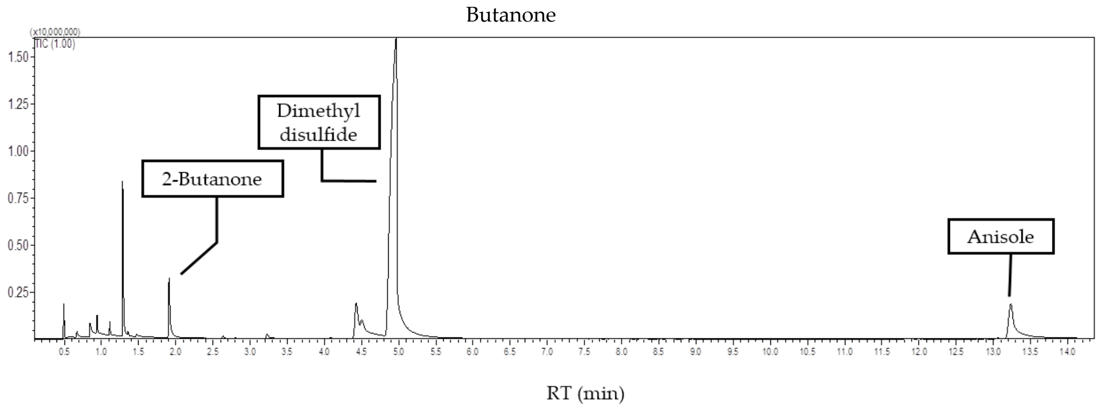

Figure S9. The GC chromatogram of VOCs emitted by *Serratia rubidaea* (A) or *Serratia marcescens* (B). Both strains were cultivated on LB agar. The peaks without labels represent components that were also detected in the medium control.

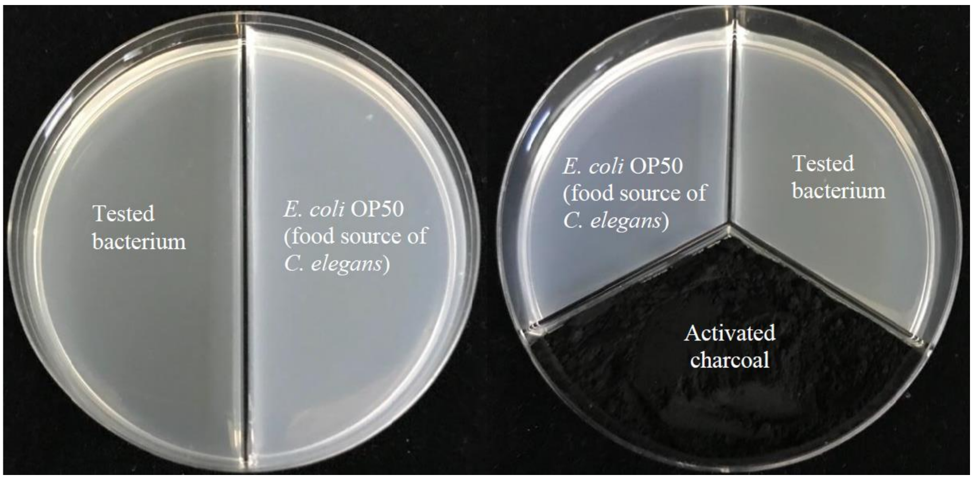

Figure S10. Display of bacteria in the fumigant activity test using two- or three-compartment Petri dishes. A chunk of agar with *C. elegans* was placed later onto the center of the compartment on which *E. coli* OP50 was grown.
